# Receptor-mediated endocytosis by Megalin: Exploring its role in ligand interaction and disease mechanisms

**DOI:** 10.1016/j.gendis.2025.101891

**Published:** 2025-10-24

**Authors:** Xian Zhang, Zhijun Zhang

**Affiliations:** a Department of Neurology in Affiliated Zhongda Hospital and Jiangsu Provincial Medical Key Discipline, School of Medicine, Research Institute of Neuropsychiatry, Key Laboratory of Developmental Genes and Human Disease of Ministry of Education, Southeast University, Nanjing, Jiangsu 210096, China; b Shenzhen Key Laboratory of Precision Diagnosis and Treatment of Depression, Department of Mental Health and Public Health in Faculty of Life and Health Sciences of Shenzhen University of Advanced Technology, The Brain Cognition and Brain Disease Institute of Shenzhen Institute of Advanced Technology, Chinese Academy of Sciences, Shenzhen, Guangdong 518055, China

**Keywords:** Endocytosis, LRP2, Megalin, Nephrotoxicity, Therapeutics

## Abstract

This review comprehensively summarizes the interaction mechanisms between Megalin and several key ligands, including calcium ions, gentamicin, ApoE, ANKRA2, FVIII, TTR, STC1, RAP, and MMP-9, focusing on the specific amino acid binding sites involved. The analysis highlights the structural basis of these interactions and their clinical relevance, particularly concerning diseases such as nephrotoxicity, Alzheimer’s disease, metabolic disorders, and renal pathologies. This review comprehensively summarizes the specific binding sites of Megalin with its ligands and explores the mechanisms, including protein reabsorption, blood coagulation, and neuroprotection, by integrating the results of animal studies and human clinical studies. This review proposes a theoretical framework for designing therapeutic strategies that target the binding sites of Megalin with its ligands. Gene editing technology and monoclonal antibody therapy aim to regulate Megalin receptor–ligand interactions to achieve therapeutic effects on related diseases.

## Introduction

Megalin is a multi-functional receptor that is critical in various physiological processes, including renal reabsorption, blood coagulation, and neuronal function. This article explores the intricate mechanisms underlying the binding of ligands to Megalin, focusing on the structural interactions that facilitate these processes and their clinical implications. Megalin’s ligand-binding repeats are crucial for calcium-dependent interactions, where calcium ions (Ca^2+^) enhance ligand affinity, stabilize the receptor’s structure, and modulate electrostatic interactions at the receptor-ligand interface. These binding mechanisms are fundamental to Megalin’s involvement in a range of cellular functions, contributing to its diverse physiological roles.

The clinical significance of these interactions is particularly evident in renal pathology, where the accumulation of drugs, such as gentamicin, through its interaction with Megalin, leads to nephrotoxicity and hearing loss, underscoring the need for therapeutic strategies that address receptor-ligand binding. Understanding the binding kinetics and structural dynamics of these interactions opens the door to novel drug development strategies targeting the Megalin receptor. For instance, designing inhibitors or small molecules that block harmful ligand-Megalin interactions could mitigate nephrotoxicity and auditory damage caused by drugs like gentamicin.

Moreover, in the context of neurodegenerative diseases, such as Alzheimer’s disease (AD), Megalin’s ability to clear amyloid-beta (Aβ) through receptor-ligand binding presents a promising avenue for therapeutic intervention. By modulating this interaction, it may be possible to enhance Aβ clearance from the brain, thus potentially reducing the progression of AD. Additionally, the interactions of Megalin with ligands like ankyrin repeat domain-containing protein 2 (ANKRA2), coagulation factor VIII (FVIII), and transthyretin (TTR) could pave the way for targeted therapies in treating metabolic disorders, coagulation abnormalities, and neurodegenerative conditions.

In terms of translational applications, the understanding of Megalin’s ligand-binding mechanisms is pivotal in advancing drug discovery and therapeutic development. Potential strategies include gene editing approaches to modify receptor expression, the design of monoclonal antibodies that can specifically modulate receptor-ligand interactions, and the development of small-molecule drugs that can either enhance or block Megalin-mediated processes. These advancements could lead to groundbreaking treatments for a variety of clinical conditions, including kidney diseases, neurodegenerative disorders, and metabolic imbalances.

## General characteristics of Megalin

### Structure of the gene encoding Megalin

**Figure 1:**
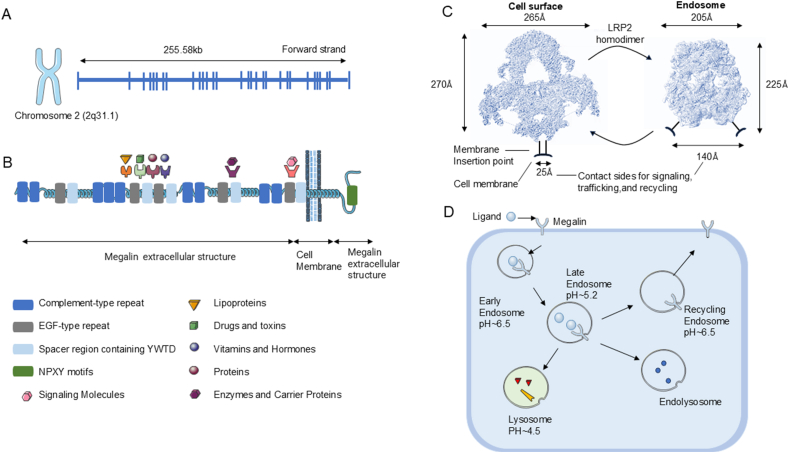
Megalin structure and functions. **(A)** The *LRP2* gene is located on chromosome 2 (2q31.1), spanning 255.58 kb, and is shown on the forward strand. **(B)** The schematic representation of Megalin protein structure includes various extracellular domains, such as complement-type repeats, growth factor repeats, spacer regions containing YWTD motifs, EGF-type repeats, and NPXY motifs, which interact with ligands like lipoproteins, drugs, toxins, vitamins, hormones, proteins, enzymes, carrier proteins, and signaling molecules. **(C)** The conformational changes of Megalin homodimers are at the cell membrane and in endosomes. On the left, a surface-bound Megalin homodimer is depicted, with dimensions of 265 Å × 270 Å and a membrane insertion point located 25 Å away from the cell membrane. On the right, a Megalin homodimer within an endosome is shown with reduced dimensions of 205 Å × 225 Å, highlighting interaction sites associated with signaling, transport, and recycling. The arrows indicate the trafficking process between the cell surface and endosomes. **(D)** The figure illustrates the intracellular trafficking process of ligands upon binding to the Megalin. Ligands are internalized via Megalin and initially enter early endosomes with a pH of approximately 6.5. As the endosomes mature, they transition into late endosomes with a lower pH (∼5.2). A portion of the endosomal contents is subsequently rerouted to recycling endosomes (pH ∼6.5), while other components proceed to endolysosomes and lysosomes (pH ∼4.5). The arrows indicate the trafficking pathway of ligands in association with Megalin.

The low-density lipoprotein receptor-related protein 2 (*LRP2*) gene is located at 2q37 on chromosome 2 and includes a 5′ flanking region of approximately 108,000 base pairs and a similarly sized 3′ flanking region.^1^ The *LRP2* gene comprises 50 exons and encodes the Megalin protein, which consists of approximately 4600 amino acids.^2^ The *LRP2* gene is situated near other genes that are involved in lipid metabolism and protein trafficking.^3^ A deeper comprehension of the genomic structure of *LRP2* will enhance our understanding of the role of Megalin in a variety of physiological processes, especially its role in kidney function,^4^ neurological diseases,^5^ and endocytosis-related disorders^6^ (Fig. 1A).

## *LRP2* gene polymorphism

The *LRP2* gene has been identified with several single-nucleotide polymorphisms that affect the physiological effects and disease associations of Megalin,^7^ including rs3755166 (G/A), rs4668123 (C/T), and rs2075252 (C/T), which have been extensively studied in metabolic and cardiovascular health.^8^ Specifically, the rs2075252 (TT) genotype increases the odds of central obesity in men, while the rs2075252 (CT) genotype reduces the rate of abdominal fat accumulation. The frequency of these polymorphisms varies between populations, with certain alleles being more common in certain ethnic groups, and this variability contributes to differences in disease susceptibility, particularly metabolic syndrome.^9^ The rs3755166 variant has been found in European and Asian populations, suggesting an effect on vitamin D homeostasis and fat distribution.^10^

The *LRP2* gene is tested in 2183 subjects to explore its potential association with AD.^11^ The results showed that the rs3755166 (G/A) polymorphism within the *LRP2* gene promoter was associated with AD risk (G/A), and the frequency of the AA genotype of rs3755166 was significantly higher in AD patients.

## Protein structure of Megalin

Megalin and the low-density lipoprotein receptor (LDLR) family share the same structural motif.^12^ Megalin consists of ligand-binding repeats, epidermal growth factor (EGF) repeats, the YWTD domain, a single transmembrane domain, and a short cytoplasmic domain. The structure of Megalin is modular, with multiple functional domains that mediate its diverse roles in ligand binding and endocytosis (Fig. 1B).

The ligand-binding repeats of Megalin are predominantly accountable for the binding of a diverse array of ligands, including vitamins, lipoproteins, and protease–inhibitor complexes. The structural changes of Megalin homodimers between the cell surface and endosomes lead to the important roles of Megalin in signal transduction, trafficking, and recycling^3^ (Fig. 1C and D). The EGF-like YWTD domains could maintain the structural stability of the receptor and facilitate ligand binding. These domains also serve as a key structural element that supports the receptor’s endocytic function. The intricate structure of Megalin, encompassing essential motifs for ligand binding, internalization, and phosphorylation, allows Megalin to carry out vital physiological functions across various organs. Furthermore, polymorphisms in Megalin can significantly impact disease outcomes. A deeper understanding of these genetic variations could provide valuable insights into potential therapeutic approaches for diseases linked to Megalin dysfunction.

## Megalin and ligands

Megalin is expressed in many organs of the human body,^13^ where it forms a complex with Cubilin to mediate the endocytosis of a variety of ligands^14^ (Table 1). Many ligands bind to Megalin, encompassing carrier proteins,^15^^,^^16^ hormones,^17^, ^18^, ^19^ enzymes,^20^ and other molecules. Key ligands include transcobalamin-vitamin B12, insulin, apolipoproteins, EGF, and various enzymes like plasminogen activator inhibitor-1 (PAI-1),^21^ lipoprotein lipase,^22^ plasminogen,^23^ and β-amylase.^24^ Additionally, Megalin binds to several drugs and toxins, including aminoglycosides, polymyxin B, and trichosanthin.^25^ Notably, several ligands, such as aprotinin, apolipoprotein E (ApoE), lipoprotein lipase, lactoferrin, and receptor-associated protein (RAP), bind within a specific region that extends from amino acid 1111 to 1210.^26^ However, some ligands, including RAP,^27^ likely interact with additional binding sites. Beyond binding proteins, Megalin also interacts with numerous calcium ions (Ca^2+^),^28^ which are indispensable for binding other ligands. These functions position the Megalin system as a core regulator of renal metabolic balance,^13^ with defects leading to genetic tubular disorders, such as Fanconi syndrome.^29^

**Table 1:** The binding mechanisms, pathological associations, and therapeutic strategies of Megalin ligands.

Ligand	Key binding mechanism	Pathological organs/diseases	Therapeutic implications	Reference
Calcium	Ca^2+^ pocket: Asp1209, Asp1213, Asp1219, Glu1220, Tyr1206, Val1211; stabilizes disulfide bonds (Cys1188-Cys1201, *etc*.)	Kidney (proteinuria, phronic kidney disease, Heymann nephritis); brain (neurodegeneration)	Engineered Ca^2+^ mimetic peptides (preclinical)	40
Gentamicin	π-alkyl (Trp1126) + salt bridges (Asp1129, Asp1131, Asp1133); H-bond (Ser1135)	Kidney (nephrotoxicity); inner ear (ototoxicity)	Cilastatin (competitive inhibitor, rodent models); Megalin CRISPR knockout (HK2 cells)	46
STC1	Tri-leucine motif (Leu12-Leu13-Leu14) binds the Megalin signal peptide	Kidney (Fanconi syndrome); system (Donnai-Barrow syndrome, Lowe syndrome)	Peptide inhibitors blocking STC1-Megalin (preclinical)	55
ApoE/βVLDL	Cluster II (aa 1111–1210); hydrophobic pocket (Val1178/Phe1182/Leu1190) + electrostatic (Aβ Lys28-Asp1193/Glu1195)	Brain (Alzheimer’s disease, blood–brain barrier dysfunction); choroid plexus (Aβ clearance)	Humanized AC10 antibody; AAV9-LRP2 overexpression (APP/PS1 mice); Cryo-EM-guided inhibitors	26
ANKRA2	“Inverted lock-and-key”: PxLPxI/L motif binds to ANKRA2 concave surface (Tyr89/Phe102/Trp115)	Systemic (potential link to 3M syndrome via CCDC8/OBSL1)	–	64
FVIII	Dual-domain: A2 domain (Arg489/Arg491) salt-bridges with Asp1217-Glu1220 triad	Liver (hemophilia A); plasma (FVIII homeostasis)	–	68
TTR	N-terminal epitope (G14-K15-H16-T17-A18) binds to Cluster II	Brain (neuroprotection, AD); kidney (TTR reabsorption); heart (transthyretin cardiac amyloidosis)	TTR nanobody 169F7–Nb (blocks neurogenic activity)	70
RAP	Ca^2+^-dependent binding to LBD I/III (CR clusters); Epitope aa 39-53	Kidney (Heymann nephritis)	–	26
MMP-9	O-glycosylated domain binds to Cluster II; Core 1/2 glycans	Lung (acute respiratory distress syndrome); brain (Aβ clearance in AD); kidney (proteinuria)	MMP-9 silencing (acute respiratory distress syndrome models)	76
Dab1/Dab2	NPXY-like motif (Megalin CT aa 107–136) binds to Dab2 PTB; phospho-Tyr115 shifts to Dab1	Brain (neurodevelopment); kidney (proximal tubule endocytosis, Donnai-Barrow syndrome)		80

**Figure 2:**
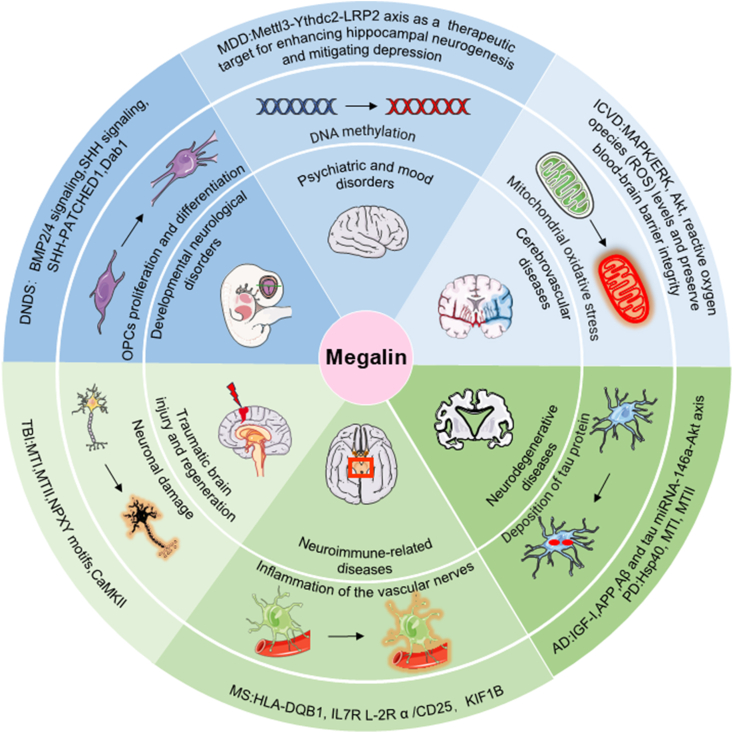
The role of Megalin in neuropsychiatric and neurological disorders. Megalin significantly influences the proliferation and differentiation of oligodendrocyte precursor cells (OPCs), which are crucial for addressing both developmental and neurological disorders. Furthermore, Megalin plays a pivotal role in regulating the major depressive disorder (MDD) pathway, particularly through its relationship with DNA methylation, offering potential therapeutic targets for enhancing hippocampal neurogenesis. Additionally, this receptor impacts mitochondrial oxidative stress and cardiovascular health by modulating reactive oxygen species (ROS) levels, maintaining blood–brain barrier integrity, and addressing related conditions, such as ischemic cardiovascular diseases (ICVDs). Megalin also participates in neuroimmune responses to vascular nerve inflammation and diseases like multiple sclerosis (MS), contributing to the associated neuroinflammatory environment. In the context of neurodegenerative diseases, particularly AD, Megalin is involved in tau protein deposition and regulates critical gene pathways, including those related to insulin-like growth factor 1 (IGF-1), amyloid precursor protein (APP), and tau mRNA. Furthermore, in traumatic brain injury (TBI) repair, Megalin influences neuronal damage and regeneration processes, which are modulated by signaling pathways such as the NPXY motifs and CaMKII.

Although Megalin’s role in the kidney has been well studied, its role in the nervous system has not been answered in detail. Recent studies have highlighted unique expression patterns and potential functions of Megalin within the central nervous system. For example, Megalin is found to be expressed in choroid plexus epithelial cells,^30^ where Megalin may mediate the endocytosis of metabolic waste products from cerebrospinal fluid,^31^ contributing to the regulation of brain homeostasis.^32^ In endothelial cells, Megalin exchanges substances between blood and brain tissue. The abnormal expression of Megalin affects the clearance of neurotoxic substances.^33^ Megalin is also expressed in neurons and glial cells^34^^,^^35^ and is closely related to synaptic plasticity. These findings suggest that Megalin functions not merely as a renal scavenger receptor but also plays a significant role in neurological diseases. For example, reduced Megalin expression was observed in the choroid plexus of AD patients, which may be associated with impaired clearance of Aβ in the cerebrospinal fluid.^36^ Conversely, the up-regulation of Megalin in the blood–brain barrier after intracerebral hemorrhage may play a neuroprotective role by promoting the clearance of hemoglobin degradation products.^37^ Although research in this area is still in its early stages, the cell-specific expression and functional diversity of Megalin in the central nervous system provide new insights into the potential mechanisms of neurological diseases (Fig. 2).

## The distinctive role of LRP2 in the LDLR receptor family

Among the members of the low-density lipoprotein receptor (LDLR) superfamily, Megalin stands out for its unique molecular size, structural complexity, and specific functions. While most family members, such as LRP1, LRP5/6, and very low-density lipoprotein receptor (VLDLR), share a common modular structure,^38^ LRP2 stands out for its clustered organization of four ligand-binding A domains as revealed by recent cryo-electron microscopy studies.^12^ Functionally, LRP2 uniquely mediates high-volume endocytosis and transcytosis in absorptive epithelial cells, particularly in the renal proximal tubules and choroid plexus. Unlike classic low-density lipoprotein receptor-related proteins (LRPs) that primarily regulate signaling, such as Wnt signaling regulated by LRP6, LRP2 focuses on bulk reuptake and clearance, processing over 75 ligands, including vitamin D-binding protein, albumin, sonic hedgehog (SHH), and amyloid-β. It exhibits remarkable pH-dependent conformational dynamics, a property not observed in other LRPs, which facilitates ligand release within acidic endosomal compartments. LRP2 cytoplasmic tail contains unique trafficking motifs, such as NPxY, dileucine, and PDZ binding sites, enabling precise sorting and recycling. Importantly, mutations in LRP2 cause Donnai-Barrow syndrome, highlighting its critical role in development. In summary, LRP2’s size, binding breadth, epithelial localization, and trafficking specializations make it a functionally and structurally distinct member of the LRP family, with key implications for renal physiology, neurodevelopment, and disease.^39^ LRP2 is distinguished from other LDLR family members by its unusually large size, multiclustered LA repeat structure, pH-responsive kinetics, broad ligand specificity, epithelial localization, complex cytoplasmic trafficking signaling, and disease-specific functional roles. These properties place LRP2 at a unique functional intersection of endocytic clearance, signaling regulation, and epithelial tissue homeostasis, justifying its designation as an LRP with distinct physiological and pathological significance.

## Specific mechanisms of receptor-ligand binding of Megalin

Megalin can recognize and bind to a variety of structurally and functionally diverse ligands, including ApoE, TTR, FVIII, gentamicin, stanniocalcin 1 (STC1), and matrix metalloproteinase 9 (MMP-9). Under physiological conditions, Megalin mediates protein reabsorption, calcium and phosphate metabolism regulation, and neuroprotective signaling. Its multiligand binding capacity relies on precise coordination between its cysteine-rich domain and the calcium-binding site. Under pathological conditions, Megalin forms complexes with toxic molecules (such as gentamicin), leading to nephrotoxicity and ototoxicity. In AD, MEGALIN’s regulation of Aβ clearance is impaired, and its functional impairment is closely associated with conditions such as Donnai-Barrow syndrome and Fanconi syndrome. Emerging research, through structural analysis, mutational analysis, and clustered regularly interspaced short palindromic repeats (CRISPR) intervention, has revealed the mechanisms linking ligand-specific recognition to disease, indicating that Megalin is a key hub connecting metabolic homeostasis, toxic responses, and neurodegenerative diseases, and possesses significant therapeutic potential.

## Calcium

Structural analysis using nuclear magnetic resonance (NMR) and asymmetric realization of integrated information (ARIA) modeling reveals that the Megalin domain contains three critical disulfide bonds (Cys1188-Cys1201, Cys1195-Cys1214, Cys1208-Cys1223) and a calcium-binding pocket coordinated by Asp1209, Asp1213, Asp1219, Glu1220, Tyr1206, and Val1211.^40^ This unique configuration differentiates it from other LDLR family members.^41^ Computational simulations suggest a 30%–50% reduction in surface positive charge density, which may contribute to ligand selectivity. Functional studies indicate that Ca^2+^ plays a dual role in structural stabilization, where calcium binding increases Megalin resistance to thermal denaturation, as shown by differential scanning fluorimetry assays.^12^ In electrostatic modulation, calcium enhances binding to anionic ligands like apolipoprotein E4, with mutagenesis of Asp1213 reducing affinity by more than 10-fold.^42^

Clinically, defects in calcium-binding are associated with renal dysfunction and drug toxicity. The lower renal Megalin mRNA levels are linked to proteinuria in patients with chronic kidney disease.^43^ In response to fluid shear stress, Ca^2+^ elicits a rapid and reversible augmentation of apical endocytosis in proximal tubule cells. This process facilitates the effective internalization of Megalin-cubilin ligands, including albumin, and thus contributes to the reabsorption of filtered proteins.^44^ This calcium-dependent mechanism regulates the binding of receptors to their ligands, potentially influencing the pathogenesis of diseases, such as Heymann nephritis.^45^ Currently, there are no studies on emerging therapeutic strategies targeting calcium-binding sites, including engineered Ca^2+^ mimetic peptides.

## Gentamicin

Gentamicin, a clinically essential aminoglycoside antibiotic, exerts its bactericidal effects by targeting bacterial ribosomes, yet its nephrotoxic and ototoxic side effects remain a major therapeutic challenge. These adverse effects are mechanistically linked to its interaction with Megalin. Utilizing NMR titration data integrated with HADDOCK-based docking simulations, researchers have identified a critical binding interface within Megalin’s ligand-binding domain. The indole ring of Trp1126 engages gentamicin’s hydrophobic core via π-alkyl interactions, while a triad of aspartate residues (Asp1129, Asp1131, Asp1133) forms salt bridges with the antibiotic’s positively charged ammonium groups. Molecular dynamics simulations further reveal transient hydrogen bonding with Ser1135, collectively stabilizing a low-affinity complex that facilitates gentamicin internalization.^46^

This receptor-mediated uptake underlies gentamicin’s organ-specific toxicity. In renal tubular cells, Megalin-dependent endocytosis leads to lysosomal accumulation of gentamicin, which disrupts mitochondrial dynamics by promoting dynamin-related protein 1 (DRP1) mediated fission.^47^^,^^48^ Simultaneously, within cochlear hair cells, the gentamicin-Megalin complexes are transported to the stereocilia. At this location, they activate reactive oxygen species,^49^ thereby initiating oxidative stress and inducing hair cell apoptosis. Preclinical models demonstrate that *LRP2* haploinsufficiency reduces gentamicin-induced hearing, highlighting Megalin’s central role in ototoxicity.^50^ Furthermore, renal aminoglycoside uptake is entirely dependent on Megalin, as mice lacking this receptor showed no accumulation of gentamicin, establishing Megalin as the primary pathway responsible for nephrotoxicity.^51^ To mitigate these adverse effects, innovative therapeutic strategies are emerging. Competitive inhibitors such as cilastatin selectively block gentamicin binding, reducing nephrotoxicity in rodent models.^52^ CRISPR/CRISPR-associated protein 9 (Cas9) knockout of Megalin in HK2 cells increased cell viability when treated with cytotoxic gentamicin, indicating suppressed Megalin function, as modified cells showed reduced gentamicin internalization and no morphological changes, compared with parent hexokinase 2 (HK2) cells, which exhibited cell death and apoptotic features.^53^ These advances underscore the translational potential of targeting the Megalin-gentamicin axis, paving the way for precision medicine approaches in infectious disease management.

## STC1

STC1 is a secreted phosphoprotein encompassing the regulation of calcium and phosphate metabolism, cell proliferation, stress response, and organ development. The expression of STC1 was modulated by a diverse array of hormonal and stress signals.^54^ A tri-leucine motif (Leu12-Leu13-Leu14) within the Megalin signal peptide directly interacted with the STC1 N-terminal signal peptide. Mutational analysis indicated that alanine substitution of these residues (L12-14A) abolished STC1 binding, while truncating the STC1 signal peptide or mutating Megalin conserved leucines (L8/L9/L11A) similarly disrupted the interaction. The STC1-Megalin complex played a key role in cellular bioenergetics in *LRP2*-knockout mouse embryonal fibroblasts.^55^ STC1 failed to enhance mitochondrial respiration or glycolysis as measured by Seahorse assays. Wild-type cells expressing mutant STC1 (L8/9/11A) showed no mitochondrial localization of STC1 and diminished metabolic responses, establishing the STC1-Megalin axis as a critical link between extracellular signals and mitochondrial metabolic reprogramming.

The disruption of the STC1-Megalin pathway was associated with a diverse range of diseases.^56^ Dysregulation of STC1-Megalin in proximal tubules was linked to Fanconi syndrome phenotypes in *LRP2* knockout mice. Therapeutically, targeting the STC1-Megalin interface offered potential interventions, as peptide inhibitors could block STC1 binding and reverse metabolic dysfunction in Donnai-Barrow syndrome and Lowe syndrome.^55^

## ApoE

ApoE and β-very low-density lipoprotein (βVLDL) are implicated in AD through their roles in Aβ metabolism and plaque formation, with ApoE contributing to Aβ clearance and influencing neurodegenerative processes. Specifically, Bell et al provided direct *in vivo* evidence using radiolabeled Aβ42-apoJ complexes in mice, demonstrating that administration of LRP2-specific antibodies significantly blocked complex clearance across the blood–brain barrier.^57^ The clearance rate of the Aβ42-apoJ complex was found to be 83% higher than that of free Aβ42, highlighting the physiological importance of apolipoprotein J (apoJ) bridging and LRP2-dependent transcytosis in Aβ removal.

In addition to the *in vivo* functional data, mechanistic studies have elucidated how LRP2 engages Aβ indirectly via apoJ. Hammad et al showed that the Aβ–apoJ complex directly binds to LRP2 through clusterin, and that this interaction is abrogated by the receptor-associated protein (RAP), a classical LRP2 antagonist.^58^ Furthermore, in LRP2-expressing cells, anti-LRP2 antibodies inhibited 59% of Aβ–apoJ complex endocytosis, and Aβ degradation was observed within the lysosomal compartment. These cellular results mechanistically reinforce the *in vivo* findings and underscore that the bridging molecule apoJ is essential for LRP2-mediated recognition and clearance of Aβ.

To further strengthen the physiological relevance of this mechanism, we have added data from recent studies reporting that LRP2 is expressed in glial cells, where it actively regulates Aβ uptake. Knockdown of LRP2 in these models abolishes stimulus-induced Aβ internalization, and treatment with clusterin up-regulates LRP2 expression while enhancing Aβ clearance.^59^ The region of amino acids 1111–1210 of Megalin constituted the key binding site for apolipoprotein E-βVLDL (apoE-βVLDL). This region selectively recognized cysteine-rich cluster II and interacted with various ligands, including apoE-βVLDL, lipoprotein lipase, and RAP, with antibody AC10 exclusively targeting this domain. Molecular dynamics simulations revealed that the hydrophobic LVFFA motif of Aβ fits into a hydrophobic pocket formed by Val1178, Phe1182, and Leu1190, while electrostatic interactions between Aβ Lys28 and Megalin’s Asp1193-Glu1195 triad stabilize the complex.^26^ Megalin-mediated Aβ clearance at the blood–brain barrier through two key pathways: transcytosis of Aβ–ApoJ complexes via clathrin-dependent endocytosis, leading to lysosomal degradation, and choroid plexus-mediated efflux of Aβ complexes with insulin-like growth factor I (IGF-I) into cerebrospinal fluid, a process disrupted in AD patients.^60^ In aged mice, Aβ clearance efficiency declined, correlating with amyloid plaque accumulation.^61^ The *LRP2* polymorphism rs3755166 (G/A) allele was associated with accelerated AD progression, particularly in APOE4 carriers.^11^ Therapeutic strategies targeting Megalin included humanized AC10 variants to enhance Aβ clearance by blocking competing ligands.^62^ AAV9-driven LRP2 overexpression restored cerebrospinal fluid Aβ42 levels and improved spatial memory deficits in APP/PS1 mice.^63^ Emerging technologies, such as cryo-electron microscopy structural insights, have revealed druggable pockets for small-molecule inhibitors, while single-cell omics and CRISPR screening provide avenues for identifying new therapeutic targets.

## ANKRA2

The ankyrin repeat family A protein 2 (ANKRA2) interacts with Megalin through a conserved Pro-x-Leu-Pro-x-Ile/Leu (PxLPxI/L) motif, utilizing an “inverted lock-and-key” binding mechanism. Structural analysis via cryo-electron microscopy showed that the concave surface of ANKRA2 (the “lock”) enveloped the PxLPxI/L motif (the “key”) on Megalin. Key interactions included hydrophobic anchoring, where Leu residues in the motif inserted into a hydrophobic pocket formed by ANKRA2’s Tyr89, Phe102, and Trp115, and electrostatic complementarity, with ANKRA2’s Asp76 and Arg93 forming salt bridges with Megalin’s Pro-Leu-Pro segment, stabilizing the complex. Mutagenesis of the PxLPxI/L motif reduced binding affinity, confirming its functional importance.^64^

The ankyrin repeat domain of ANKRA2 recognized a PxLPxL/I motif, which was present in multiple proteins, including Megalin, several histone deacetylases, and regulatory factor X (5RFX5). Structural and binding analyses indicated that the ankyrin repeat domain of ANKRA2 was capable of binding to the PxLPxL motif located in the C-terminal region of coiled-coil domain containing 8 (CCDC8).^65^ The interaction between ANKRA2 and CCDC8 underscored ANKRA2 as a pivotal protein partner implicated in the functional regulation of CCDC8. Mutations in the CCDC8 gene were associated with 3M syndrome, a disorder characterized by short stature accompanied by facial and skeletal anomalies. Additionally, ANKRA2 interacted with obscurin-like 1 (OBSL1) to form a complex with cullin 7 (CUL7), an E3 ubiquitin ligase. These findings established a connection between ANKRA2 and 3M syndrome,^66^ suggesting that ANKRA2 may have a role in novel regulatory mechanisms that involve histone deacetylases and RFX7.

## FVIII

FVIII interacts with Megalin through dual-domain recognition. The primary binding occurred via the FVIII A2 domain (residues 484–509), where surface plasmon resonance assays showed high-affinity binding between FVIII’s Arg489/Arg491 and Megalin’s Asp1217-Glu1220 triad, with alanine substitution of Arg489 (R489A) reducing binding.^67^^,^^68^

Hemophilia A represents an X-linked recessive hemorrhagic disorder. Research had elucidated that the Megalin receptor exerted a significant influence on the hepatic clearance of FVIII. In murine models lacking the LDLR, elevated levels of plasma FVIII were observed, signifying that Megalin was indispensable for the maintenance of FVIII homeostasis. Furthermore, combined deficiency of LDLR resulted in a more pronounced increase in FVIII levels compared with mice lacking only *LRP*2, suggesting a compensatory mechanism between these receptors in FVIII clearance. These findings underscored the complex interplay between LDLR and LRP in regulating FVIII levels.^69^

## TTR

TTR interacts with Megalin through a critical interface formed by TTR N-terminal residues 14–18 (G14-K15-H16-T17-A18), which engage Megalin’s ligand-binding cluster II.^70^ Mutagenesis of K15 (K15N) abolished binding, confirming its importance. Additionally, TTR-specific nanobody 169F7–Nb competed with Megalin binding by occupying the 14–18 epitope, blocking TTR neurogenic activity in cortical neuron cultures. This interaction was significant in disease mechanisms, such as in transthyretin amyloidosis, where misfolded TTR aggregates disrupted Megalin-mediated clearance, leading to amyloid deposition in cardiac and neuronal tissues.

Research findings suggested that TTR facilitated neurite outgrowth and conferred neuroprotection by interacting with Megalin. In hippocampal neurons, recombinant TTR was demonstrated to promote neurite growth. This interaction activated the mitogen-activated protein kinase (MAPK) and protein kinase B (Akt) pathways, and under excitotoxic conditions, TTR stimulation was shown to rescue neurons from cell death and prevent neurite loss in Megalin-deficient neurons, thus emphasizing the critical role of Megalin receptors in mediating the neuroprotective effects of TTR.^71^

In addition to the nervous system, Megalin also reabsorbs TTR in the kidneys. Studies showed that TTR bound to Megalin and promoted its internalization into renal cells. This interaction suggested that Megalin was involved in the renal processing of TTR, which may have an impact on renal function and TTR clearance from the blood.^72^

## RAP

Heymann nephritis, serving as a rodent model, is distinguished by the deposition of subepithelial immune complexes along the glomerular basement membrane, with the ectodomain of Megalin acting as the primary autoantigen. The extracellular domain of Megalin (aa 1114–1250) directly bound circulating antibodies to form immune complexes, and the interaction between Megalin and the RAP was investigated.^26^ Specifically, the cryo-electron microscopy analysis revealed that RAP bound to two specific regions on Megalin, corresponding to Lewy body dementia (LBD I) and Lewy body dementia (LBD III). These binding regions were associated with the complement-type repeat (CR) clusters. RAP’s binding involved multiple positively charged patches on its structure, which interacted with the CRs of Megalin through typical Ca^2+^ dependent interactions.^12^ Co-immunoprecipitation assays revealed that RAP did not colocalize with these immune complexes, confirming RAP’s non-essential role in the pathogenesis of Heymann nephritis.^73^ Active Heymann nephritis models, induced by immunization with recombinant Megalin fusion protein, showed high-titer IgG antibodies and diffuse granular immune complex deposits.^74^ Previous investigations had localized the principal ID-inducing antibody binding site to the initial 86 amino acids of RAP (RAP1-86). Collectively, these results identified the ID-inducing epitope of RAP as being situated within the amino acid sequence spanning from 39 to 53. Although the precise mechanism underlying the interaction between RAP and Megalin was not explicitly delineated in this study, it was likely to involve conformational interactions that facilitated the formation of immune complexes capable of inducing glomerular pathology.^75^

## MMP-9

Megalin serves as a pivotal receptor for MMP-9, specifically identifying its hemopexin-like (PEX) domain via a dual-binding mechanism. The O-glycosylated (OG) domain of MMP-9, which is composed of 12 Thr/Ser-Xxx-Xxx-Pro repeats, interacts with Megalin’s ligand-binding cluster II, and core 1 (Galβ1-3GalNAcα1-) and core 2 (GlcNAcβ1-6[Galβ1-3]GalNAcα1-) O-glycans, with glycan array analysis demonstrating high-affinity binding.^76^ Clinical research reported that MMP-9 was involved in the metabolism and clearance of Aβ, a key component of senile plaques. The expression of MMP-9 in astrocytes was modulated by amyloid-associated proteins, particularly apolipoprotein E, which, in conjunction with Aβ, induced MMP-9 expression. Additionally, MMP-9 interacts with cargo receptors, such as Megalin and LDLRD1, facilitating the endocytosis and degradation of the enzyme. The study suggested that these regulatory mechanisms were impaired in astrocytes from AD patients, potentially contributing to the exacerbation of Aβ accumulation and progression of AD pathology due to defective MMP-9 clearance and regulation.^77^ Another investigation demonstrated the molecular mechanisms responsible for the down-regulation of Megalin expression in acute respiratory distress syndrome, specifically emphasizing the role of MMP-9. This study revealed that transforming growth factor-beta (TGF-β) treatment induced the shedding of Megalin and its regulated intramembrane proteolysis, resulting in an augmentation of the intracellular accumulation of the Megalin C-terminal fragment, which subsequently led to the transcriptional down-regulation of Megalin. MMP-9 was identified as a crucial mediator in the TGF-β-induced shedding of Megalin. The silencing of MMP-9 stabilized Megalin at the cell surface and restored normal albumin transport. Moreover, the direct interaction between Megalin and MMP-2/MMP-14 indicated that these MMPs served as novel sheddases of Megalin, contributing to the pathophysiology of acute respiratory distress syndrome. These findings offered insights into the regulatory role of MMP-9 in Megalin processing and presented potential avenues for therapeutic interventions in acute respiratory distress syndrome.^78^ The study investigated the role of neutrophil gelatinase-associated lipocalin (NGAL)/lipocalin-2 in breast cancer aggressiveness using a mouse model. MMP-9, a matrix metalloproteinase, was one of the markers examined concerning lipocalin-2 expression. Despite observing high expression of lipocalin-2 in tumors from wild-type mice, no significant difference in MMP-9 expression or other markers between the wild-type and lipocalin-2-deficient mice was noted.^79^

## Dab1/Dab2

Disabled-1 (Dab1) and Disabled-2 (Dab2) are adaptor proteins involved in neurodevelopment and endocytic pathways, modulating cell migration, positioning, and signal transduction through interactions with receptors and kinases. The cytoplasmic tail (CT) of Megalin (residues 107–136) regulated receptor trafficking through conserved sorting motifs, including the NPXY-like motif, which bound to Dab2 via its phosphotyrosine-binding (PTB) domain.^80^ Functional divergence between Dab1 and Dab2 dictated tissue-specific regulation, with Dab1 dominating in neuronal cells through reelin signaling and Src family kinases (SFK), whereas Dab2 primarily regulated Rab5/Rab7 endosomal trafficking in renal proximal tubules.^81^ Phosphorylation of Tyr115 by Fyn kinase shifted adaptor preference from Dab2 to Dab1, enhancing phospho-Tyr115-Dab1 affinity by sevenfold.^82^

Employing RNA sequencing and CRISPR/Cas9 gene knockout technology identified proximal tubule (PT)-specific transcriptional alterations that were directly associated with the knockout of *LRP2* and *Dab2* in a cell culture model. Among these, the knockout of *LRP2* exerted the most significant transcriptional effect, as nearly all genes with altered expression in *Dab2* knockout cells also displayed changes in *LRP2* knockout cells. These changes were congruent with the pathological alterations observed in knockout mice and patients with Donnai-Barrow syndrome. Moreover, the disparities in transcriptional profiles between *LRP2* knockout cells suggest that aberrant receptor localization may contribute to the transcriptional changes by altering spatial signaling, which in turn disrupts PT endocytic function. In summary, these findings revealed the role of Megalin as a master regulator that orchestrates ion transport, metabolism, and endocytosis in the PT. These changes were consistent with the pathological alterations observed in knockout mice and patients with Donnai-Barrow syndrome.^83^

## Ligands of Megalin

As one of the most versatile scavenger receptors in mammals, Megalin’s functional diversity arises from the dynamic conformational changes of its four extracellular cluster domains (CL1–CL4) and its broad ligand compatibility.^84^ While current research has unveiled precise interaction sites for a few ligands, over 80% of Megalin ligands lack atomic-level binding mechanism data. Notably, these ligands have been identified as functional partners of Megalin through several lines of evidence, including spatial colocalization mechanisms, where immunofluorescence shows Megalin co-depositing with lipoproteins at sites of tubular injury in diabetic nephropathy; functional dependencies, such as iron metabolism dysregulation and hormonal imbalances observed in *LRP2* knockout mice; and intervention responsiveness, where anti-Megalin antibodies block the tubular uptake of aminoglycoside antibiotics, reducing nephrotoxicity. Based on biological function and pathological relevance, these ligands can be classified into six categories, with their potential binding modes likely following universal principles, such as “charge complementarity”, “hydrophobic motif embedding”, or “chaperone-mediated synergism”. This section will systematically review the functional evidence for each ligand class and hypothesize their potential binding domains, providing a prioritized framework for mechanistic exploration.

## Vitamin-binding proteins

Megalin mediates the transport and reabsorption of various vitamin-binding proteins in the human body. These include folate-binding protein (FBP),^85^ retinol-binding protein (RBP),^4^^,^^86^ intrinsic factor (IF),^87^ transcobalamin,^27^ and vitamin D-binding protein (VDBP).^88^^,^^89^ Within the intestinal tract, cobalamins specifically bind to the intrinsic factor (IF) to facilitate their absorption. This process is subsequently followed by endocytosis, which is mediated by cubilin. Megalin may potentially play an assisting role in the uptake of the IF-cobalamin complex. Following absorption, vitamins are transported within the bloodstream and are taken up by tissues via their respective carrier proteins.

In addition, a deficiency in Megalin results in developmental defects, which may be caused by a vitamin deficiency or impaired lipid transport. VDBP, which is responsible for transporting vitamin D, also interacts with Megalin in the kidneys, and the reabsorption of VDBP helps activate vitamin D, which is essential for calcium regulation. Megalin deficiency resulted in reduced levels of vitamin D metabolites and severe bone defects.^90^ Overall, Megalin and cubilin contribute to maintaining vitamin and nutrient homeostasis, ensuring proper vitamin D activation and transport, and supporting embryonic and metabolic functions.

## Lipoproteins

Megalin exerts a significant role in cholesterol transport, especially during the process of development, in cooperation with its coreceptor, cubilin. Megalin served as a receptor for multiple lipoproteins, such as apoE and apoJ/clusterin.^91^ Additionally, Megalin serves as the receptor for apolipoprotein M (apoM),^92^ VLDLs, and LDLs that are secreted by the liver and kidneys. In conjunction with cubilin, Megalin also internalizes apolipoprotein A-I (apoA-I)^93^ and apolipoprotein A-II (apoA-II),^94^ which serve as structural constituents of high-density lipoproteins, thereby playing a role in the regulation of high-density lipoprotein metabolism. Genetic polymorphisms in the Megalin gene have been associated with plasma cholesterol levels and LDL cholesterol, especially among the Japanese population. This further indicates that Megalin has a systemic function in maintaining cholesterol homeostasis.^95^

## Hormones

Megalin is implicated in the endocytic uptake and transcellular transport of diverse hormones and growth factors, encompassing insulin,^96^ prolactin,^97^ EGF,^98^ sex steroids,^99^ and the thyroid pro-hormone thyroglobulin. Megalin is expressed on the luminal surface of thyroid epithelial cells and can facilitate the uptake of thyroglobulin into thyrocytes. This process competes with mechanisms that convey thyroglobulin to lysosomes for proteolytic cleavage and thyroid hormone release.

Furthermore, Megalin has been associated with the regulation of calcium homeostasis. It binds to several ligands, including calcium, VDBP, and parathyroid hormone (PTH), suggesting that Megalin may function as a calcium sensor.^90^ It has been observed that Megalin binds to calcium in the renal cortex, contributing approximately 40% of the total calcium-binding activity. Megalin also mediates the lysosomal degradation of PTH in renal proximal tubules, aiding in the regulation of the hormone’s activity by removing it from the filtrate.^100^ Interestingly, elevated Megalin expression has been observed in the thyroid glands of patients with Graves’ disease.^101^ Circulating anti-Megalin antibodies have been detected in patients with autoimmune thyroiditis.^102^ These findings indicate that Megalin plays a pivotal role in calcium and hormone homeostasis, particularly concerning thyroid and kidney function.

## Drugs

Many drugs were absorbed by Megalin in the proximal tubule, such as aprotinin,^103^ polymyxin B,^104^ and aminoglycosides,^105^ including gentamicin. This process was confirmed using knockout mice, which demonstrated a reduced uptake of gentamicin in the proximal tubules. sTβRII.Fc is a potential therapeutic agent for fibrosis and albuminuria in diabetic nephropathy. It works by targeting and reducing the effects of TGF-β1, which is involved in causing disruptions to the renal proximal tubule cell’s ability to absorb albumin. By preventing these disruptions, sTβRII.Fc may help lower albuminuria, which is a key indicator of kidney damage in diabetic nephropathy. These drugs are known to be nephrotoxic and ototoxic, with the toxicity likely arising from the accumulation of these drugs within cells due to Megalin-mediated reabsorption.^106^ The ability of Megalin to facilitate the uptake of these drugs in the kidneys contributes to their harmful effects, as it promotes cellular accumulation, leading to toxicity in both the kidneys and ears.^107^

## Signaling molecules

Megalin regulates cell signaling via receptor-mediated endocytosis. Although a specific signaling event for Megalin has not been definitively documented, Megalin cytoplasmic tail, particularly the carboxy-terminal NPXY domain,^108^ interacts with Dab2, an intracellular protein implicated in cellular growth and differentiation.^109^ This indicates that Megalin could be involved in signaling pathways analogous to those of other members of the LDL receptor family, such as the VLDL receptor, which also binds to cytoplasmic adaptors like Dab1. These interactions, in turn, regulate crucial cellular functions such as neuronal migration, which is mediated by Reelin binding to the VLDL receptor and ApoER2.^82^ The disruption of this pathway, observed in mice deficient in Dab1 or Reelin, underscores the potential signaling role of Megalin.

In addition to its role in regulating endocytosis, Megalin has been shown to undergo regulated intramembrane proteolysis, similar to other large transmembrane receptors such as Notch.^110^ Megalin intracellular domain (ICD) was released into the cytoplasm by γ-secretase activity and subsequently exerted biological effects in intracellular signaling processes.^111^ However, in contrast to the well-defined role of Notch ICD in gene regulation, the precise function of Megalin ICD release remained uncertain. The overexpression of Megalin ICD resulted in the down-regulation of Megalin and sodium-hydrogen exchanger 3 (NHE3) transcripts.^112^ Recent *in vivo* studies in mouse models had shown that expression of Megalin ICD under the control of the endogenous Megalin promoter did not produce any apparent effects on renal proximal tubule function, suggesting that release of Megalin ICD induced by ligand binding might not have significantly reduced receptor activity or protected the proximal tubule from protein overload as previously hypothesized.

Overall, Megalin appears to be implicated in a complex network of signaling pathways, especially those of calcium regulation^28^ and the Reelin signaling pathway.^113^ The proteolytic processing of Megalin may influence its function in ways that are not yet fully comprehended.

## Enzymes and inhibitors

Megalin can also be endocytosed and reabsorbed in the presence of various enzymes and inhibitors. The role of Megalin in prorenin uptake was less pronounced compared with the mannose-6-phosphate (M6P) receptor, as inhibition of Megalin expression significantly reduced prorenin binding and internalization.^114^ Megalin also interacts with angiotensinogen, the precursor to angiotensin II (Ang II), in proximal convoluted tubules of the kidney. This interaction regulated the local production of Ang II, influencing vascular tone and potentially contributing to atherosclerosis.^115^

Moreover, Megalin was shown to bind to protein kinase B (PKB) in renal proximal tubule cells,^116^ which affected cellular survival and injury responses, particularly regarding albumin. This suggests that Megalin dysfunction might contribute to diseases related to impaired protease regulation, including tissue remodeling disorders and certain cancers.^117^ Immunoglobulin light chains, when accumulated in proximal tubule cells expressing Megalin, were preferentially reabsorbed by Megalin and contributed to tubule injury and nephrotic syndrome by triggering inflammatory responses and cellular apoptosis.^118^

In summary, Megalin interacts with RAP, M6P receptor, angiotensinogen, and PKB, and Megalin promotes the reabsorption of enzymes and inhibitors and modulates signaling pathways that affect renal function and vascular health.

## Functional characteristics of Megalin and its role in diseases

In congenital disorders, Donnai-Barrow syndrome represented the most characteristic phenotype caused by monogenic mutations in Megalin. The underlying molecular mechanisms have been gradually elucidated. Mutations in Megalin disrupted the Shh signaling axis and impaired the differentiation pathways between the neural tube and the eye during embryogenesis, ultimately resulting in dominant brain malformations, retinal dysplasia, and sensorineural hearing loss, among other multisystem defects.^136^ Functional studies revealed that Megalin mutants lost high-affinity binding to critical ligands, including Shh and retinol-binding protein, while retaining partial endocytic capacity. These findings suggest that the pathogenic mechanism might involve fine-grained regulation of ligand-recognition domains and precise coupling with intracellular signaling networks.

In acquired diseases, Megalin played dual roles in the central nervous system, contributing to both neuroprotection and metabolic clearance. Its expression in the choroid plexus epithelium was critical for removing metabolic waste from the cerebrospinal fluid. A significant correlation was observed between choroid plexus structural disruption and reduced Megalin expression in AD patients, indicating that Aβ accumulation might be primarily due to impaired clearance rather than overproduction.^57^ Megalin functioned as a molecular bridge during the translocation of Aβ peptides from cerebrospinal fluid into the bloodstream, in cooperation with ApoE and other transport proteins. Mouse model studies further demonstrated that enhancing Megalin function improved Aβ clearance rates and ameliorated cognitive impairments, offering a potential therapeutic strategy for AD.^58^

In the kidney, Megalin was predominantly located at the brush border of proximal tubules, where it mediated the reabsorption of proteins, vitamins, and hormone-bound small molecules. Proximal tubule dysfunction was frequently observed in patients with chronic kidney disease and was strongly associated with reduced Megalin expression or defective ligand binding.^43^ During the progression of diabetic nephropathy, the decreased transport capacity mediated by Megalin led to the loss of essential nutrient molecules, such as transferrin, VDBP, and fat-soluble hormones, which in turn accelerated tubular atrophy and glomerulosclerosis.^84^

In conclusion, the central role of Megalin dysfunction in a wide range of diseases has become increasingly evident. Its functions in the nervous system, kidney, inner ear, and other absorptive epithelia extend beyond molecular transport to include signal integration and cell fate determination. Systematically linking Megalin molecular mechanisms to clinical manifestations not only facilitated the identification of shared pathogenic pathways across complex diseases but also provided a theoretical basis for developing precise, multisystem-targeted therapeutic strategies.

## The overlap of ligand binding sites

Megalin is a multifunctional endocytic receptor, with its primary ligand-binding sites located within four cysteine-rich complement-type repeat clusters (Cluster I-IV). While certain binding regions, such as Cluster II, have been identified, the exact amino acid residues for many ligands remain unresolved. For instance, apolipoprotein E, lactoferrin,^119^ and RAP all bind within Cluster II, and competitive binding experiments using monoclonal antibody AC10 suggest a potential spatial overlap of these ligands.^120^^,^^121^ However, it is still unclear whether they bind to the same amino acid residues. Structural studies indicate that complement-type repeats have conserved calcium-binding sites and disulfide bonds, supporting the conservation of the binding motif.^98^ For example, gentamicin binds to the 10th repeat in Cluster II, with key residues including Trp-1126 and Asp-1129.^122^^,^^123^ Whether other ligands such as ApoE share these residues remains uncertain. Cryo-electron microscopy studies further reveal Megalin dimerization and ligand binding, but comprehensive mapping of ligand-binding sites is still needed. These findings suggest that different ligands may overlap within the same cluster, but the underlying mechanisms are complex and dependent on ligand characteristics.

## Tissue and organ-specific ligands

Megalin is expressed in various tissues, including the kidney, brain, liver, and heart, and its ligands exhibit significant tissue specificity, influenced by the local microenvironment and physiological barriers. In proximal tubule epithelial cells of the kidney, Megalin binds to ligands, such as vitamin D-binding protein, retinol-binding protein, and albumin, to facilitate protein reabsorption, preventing their loss in urine.^124^ In the brain, Megalin clears Aβ through the choroid plexus and neurons, which is particularly important in AD.^125^ In the heart, Megalin interacts with renin and prorenin through the renin-angiotensin system, contributing to cardiovascular regulation.^126^ Physiological barriers, such as the blood–brain barrier and the local tissue microenvironment, determine the accessibility of ligands. For example, although albumin was reabsorbed by Megalin in the kidney, it could not cross the blood–brain barrier to interact with Megalin in the brain, and Aβ, which was normally present in the brain, was not present in the kidney microenvironment.^127^^,^^128^ If kidney-specific ligands such as VDBP were artificially introduced into the brain, they could theoretically bind to Megalin, but such inter-organ interactions were rare under normal circumstances. This tissue-specific ligand interaction reflected the adaptive functions of Megalin in different organs.

## Dependence of Megalin function on ligand binding

Current evidence suggests that all of Megalin’s functions depend on ligand binding, and no ligand-independent signaling or structural functions have been identified. As a primary endocytic receptor, Megalin requires ligand binding to initiate the formation of clathrin-coated vesicles, mediating substance uptake. Furthermore, Megalin facilitated the transport of ligands such as Ang II^129^ and stanniocalcin-1 (STC1)^55^ to mitochondria, enhancing antioxidant defenses, but this process also required ligand binding at the cell membrane. Megalin-deficient mice exhibited phenotypes, such as proteinuria and neurodegeneration, but no abnormalities were observed in other functions, such as electrolyte transport or cellular structure maintenance.^130^ For example, in the kidney, Megalin formed a complex with cubilin and amnionless, mediating the endocytosis of low-molecular-weight proteins, and the loss of this function directly led to proteinuria in Fanconi syndrome. Therefore, Megalin’s functionality was strictly dependent on ligand binding, with its primary function being multi-ligand-mediated endocytosis and transport.

## Ligand functions across different diseases

In diseases associated with Megalin dysfunction, ligand selection is highly tissue- and disease-specific, with significant differences in downstream pathways and outcome indicators (Table 2). In kidney diseases like Fanconi syndrome,^29^ the dysfunction of ligands, such as VDBP and RBP, leads to impaired endocytic function in proximal tubules, resulting in proteinuria and vitamin metabolism disturbances.^131^ In AD, reduced Megalin expression impairs Aβ clearance, leading to neurodegeneration^132^ and compromised synaptic plasticity.^133^ In major depressive disease, microglia-derived VDBP induces depression in mice by binding to the neuronal receptor Megalin, mediating the src-non-receptor tyrosine kinase signaling pathway and leading to neuronal apoptosis and synaptic damage.^134^ In cardiovascular diseases like atherosclerosis, Megalin regulates the renin-angiotensin system, contributing to inflammation and vascular remodeling.^92^ Despite the conserved endocytosis mechanism across diseases, the tissue-specific ligands result in distinctly different pathological outcomes: kidney diseases primarily manifest as renal failure, neurodegenerative diseases as cognitive impairment or Aβ deposition, and cardiovascular diseases as hypertension and plaque formation. Bone morphogenetic protein 4 (BMP4) participates in kidney disease, bone development disorders, and other related pathological processes by affecting the expression and function of Megalin and regulating cell endocytosis and signal transduction.^135^ SHH interacts with Megalin, and its absence or dysfunction due to Megalin deficiency leads to forebrain defects, likely by disrupting the endocytosis of BMP4, which down-regulates SHH expression and causes holoprosencephaly.^136^ Notably, certain ligands, such as renin, may play a role in both renal and cardiovascular diseases, but their downstream effects differ due to the unique tissue environments.^137^ This ligand-tissue-disease specificity highlights the complexity and microenvironment-dependent nature of Megalin’s functions.

**Table 2:** The abnormal binding of ligands to Megalin in diseases.

Ligands	Disease	Mechanism	Reference
Myoglobin	Acute kidney injury	Heme in myoglobin generates reactive oxygen species under oxidative stress conditions, which directly act on renal tubular epithelial cells, causing cell apoptosis and necrosis.	14
RAP	Heymann nephritis	Heymann nephritis interferes with the Megalin-mediated autoantibody attack on the foot process protein through anti-RAP.	73
TTR	Amyloidotic neuropathy	By affecting the tissue clearance of TTR and regulating the accumulation of non-fibrillar and fibrillar TTR with Megalin/Clusterin, it leads to ATTRV30M amyloid deposition.	70
TGF-β	Chronic kidney disease	Megalin transports extracellularly applied STC1, angiotensin II, and TGF-β to mitochondria via the reverse early endosome-Golgi transport pathway and Rab32. Knockout of Megalin in cultured cells impairs glycolysis and respiratory function.	78
Leptin	Metabolic syndrome and obesity	Abnormal leptin clearance led to central leptin resistance by inhibiting the hypothalamic JAK-STAT signaling pathway.	19
PAI-1	Chronic kidney disease	PAI-1 promoted collagen deposition by activating the TGF-β1/Smad pathway.	21
Trichosantin	Acute kidney injury	Trichosantin induced oxygen species and activated the NF-κB pathway.	25
Haemoglobin	Acute kidney injury, chronic kidney disease	Hemoglobin-accumulated oxidative stress caused tubular obstruction and necrosis.	36
Ca^2+^	Renal; Fanconi syndrome	Intracellular Ca^2+^ concentration promoted NF-κB pathways.	44
EGF	Chronic kidney disease	EGFR phosphorylated ERK1/2 and STAT3, and up-regulated and promoted tubulointerstitial inflammation and fibrosis.	98
Gentamicin	Acute kidney injury, chronic kidney disease	The high accumulation of gentamicin in cells caused oxidative stress, mitochondrial dysfunction, and cell death.	53
AopE-βVLDL	AD	AopE-βVLDL reduced Aβ clearance, amyloid plaque deposition, and neuronal damage.	62
TGF-β	Chronic kidney disease	TGF-β abnormally accumulates in the cytoplasm or extracellular matrix, overactivating the TGF-β/Smad signaling pathway.	106
FBP	Hyperhomocysteinemia	Folic acid-dependent homocysteine metabolism was blocked, increasing cardiovascular risk.	85
RBP	Vitamin A deficiency	RBP retinol reabsorption disorder can cause skin issues, vision loss, and immune abnormalities.	81
RBP	Renal tubular proteinuria	Megalin function defect increased RBP excretion and renal tubular damage.	81
VDBP	Rickets/osteomalacia	Vitamin D loss causes calcium-phosphorus metabolism disorder and bone mineralization disorder.	86
Insulin	DN	Down-regulated expression or abnormal function of Megalin activated EGFR and MAPK pathways.	96
Polymyxin B	Acute kidney injury	Polymyxin B accumulation activates NADPH oxidase and the NF-κB pathway.	104
Ang II	Atherosclerosis	Ang II activates nuclear factor-κB (NF-κB) through AT1R signaling and enhances monocyte adhesion and foam cell formation.	115
VDBP	Depression	Microglia-derived VDBP induced depression by binding to Megalin, mediated the SRC signaling pathway, and led to neuronal apoptosis and synaptic damage.	134

## Clinical implications of anti-Megalin antibodies

Under autoimmune or alloimmune conditions, Megalin may become a target of pathogenic autoantibodies, leading to the development of severe tubulointerstitial injury.

Studies have shown that anti-Megalin antibodies can specifically bind to Megalin receptors on the brush border of renal tubular epithelial cells, forming immune complexes that are deposited in the brush border and basement membrane of the proximal tubules.^138^ In one case series, anti-brush border antibodies were identified in patient serum that recognized Megalin and colocalized with IgG in tubular wall deposits observed in renal biopsies. The formation of antibody–antigen complexes not only interferes with the normal endocytosis of Megalin but also triggers local immune activation, initiating an inflammatory cascade and leading to proximal tubular damage.^139^

In the Heymann nephritis model and human anti-LRP2 nephropathy, IgG4 subclass antibodies predominate. While IgG4 itself does not activate complement, binding of antibodies to Megalin can cause sloughing of the brush border of renal tubular epithelial cells, cell shrinkage and necrosis, the appearance of intratubular cell debris, and the formation of granular IgG deposits beneath the basement membrane of the proximal tubules, accompanied by interstitial mononuclear cell infiltration. These pathological changes are consistent with findings in experimental models, indicating that antibody binding directly induces cytotoxicity, driving local fibrosis and renal function loss.^140^

Clinically, anti-Megalin antibody-associated diseases are more common in elderly patients, with acute kidney injury and progressive proteinuria as the main manifestations. Pathological features include significant proximal tubular damage and IgG deposition. Studies have found that the range of Megalin epitopes recognized by antibodies in patient serum correlates with disease severity: antibodies covering multiple Megalin domains often predict more severe renal damage.^141^

Prognostically, anti-Megalin antibody-associated diseases are associated with poor clinical outcomes. Retrospective studies have demonstrated that nearly half of affected patients progress to end-stage renal disease or death if left untreated. However, early initiation of immunosuppressive therapy, particularly with glucocorticoids and B-cell-depleting agents such as rituximab, has been shown to improve remission rates significantly.^142^ Therefore, prompt detection of anti-Megalin antibodies, coupled with targeted immunomodulatory interventions, is crucial for improving renal survival and overall patient prognosis.

## Soluble Megalin as a biomarker in diabetic and chronic kidney disease

Studies have shown that in patients with type 2 diabetic nephropathy, urinary levels of both A- and C-type Megalin increase significantly with disease progression and are closely associated with a decline in estimated glomerular filtration rate (eGFR).^143^ Even in patients with normal urinary albumin levels, urinary C-type Megalin is significantly elevated compared with healthy controls.^144^ This suggests that it is highly sensitive to early kidney damage. Further cohort studies have found that patients with elevated baseline urinary A- or C-type Megalin levels have a significantly accelerated rate of subsequent eGFR decline. This association remains significant even after adjusting for traditional biomarkers such as urinary albumin and N-acetyl-beta-d-glucosaminidase (NAG). Another cohort study of 752 patients with diabetes showed that baseline urinary C-Megalin levels were strongly associated with the risk of developing microalbuminuria during follow-up, with the strongest association observed in patients with normal or low baseline urinary albumin levels.^145^

Similarly, urinary C-Megalin levels were positively correlated with proteinuria in patients with chronic kidney disease.^146^ This further suggests that soluble Megalin levels can reflect the extent of kidney damage. These findings support the potential of using A- and C-type soluble Megalin as independent biomarkers for the early diagnosis and prognosis of kidney disease. However, some studies have failed to find such an association. For example, a cross-sectional study from India showed that urinary Megalin levels were higher in non-diabetic controls than in diabetic patients, and no significant correlation was observed between urinary Megalin and urinary protein or eGFR in diabetic patients.^147^

This discrepancy may be due to factors such as sample characteristics, detection methods, such as failure to distinguish between different Megalin fragments, or cohort size. When synthesizing the results of different studies, the heterogeneity of available data should be noted. Future large-scale, multicenter, prospective studies using standardized detection protocols are needed to further validate the validity and clinical significance of type A and type C soluble Megalin as independent diagnostic/prognostic markers. In summary, current clinical evidence suggests that urinary type A and type C Megalin levels can reflect the severity of kidney disease and predict disease progression. However, their practical application requires further validation.

## Conclusions and future perspectives

Megalin is a multifunctional receptor involved in nutrient transport, lipoprotein metabolism, hormone signaling, and immune modulation. It plays a critical role in reabsorbing proteins in the kidney, clearing Aβ in the brain, regulating coagulation in the cardiovascular system, and responding to drug toxicity. Its calcium-dependent structure allows versatile ligand binding, supporting diverse physiological functions and disease mechanisms. Megalin’s functions are essential for maintaining cellular and organ-level homeostasis.

Future research should focus on deciphering the unresolved binding mechanisms of Megalin ligands, particularly those involving vitamin-binding proteins, lipoproteins, and other critical molecules. Sophisticated methodologies, including cryo-electron microscopy and molecular dynamics simulations, can be utilized to investigate the dynamic conformational alterations that occur during ligand binding and to clarify the structural foundation of these interactions. Additionally, understanding the calcium-dependent regulatory network of Megalin, beyond ligand binding, will help clarify its role in cellular signaling, including interactions with co-chaperone proteins like RAP.

Integrating structural biology, computational chemistry, and clinical data will be essential for establishing a dynamic database of Megalin-ligand interactions. Such integration can drive discoveries, particularly in the identification of novel Megalin ligands and optimizing binding affinity for therapeutic purposes. Furthermore, leveraging artificial intelligence and machine learning to predict new ligand interactions and enhance drug design could accelerate the development of novel therapeutic compounds aimed at modulating Megalin activity.

The complexity of Megalin, as a “molecular hub” in various physiological and pathological processes, is far from fully understood. Its multifunctionality in nutrient transport, immune modulation, and signaling pathways provides a wide scope for future research. With ongoing advancements in cross-disciplinary collaboration and technological innovations, the barriers to understanding its mechanisms and translating this knowledge into clinical practice can be overcome. This will open up new avenues for the precision treatment of metabolic diseases, neurodegeneration, and renal dysfunction, providing a promising future for therapeutic strategies targeting Megalin.

## CRediT authorship contribution statement

**Xian Zhang:** Writing – original draft. **Zhijun Zhang:** Supervision, Funding acquisition.

## Funding

This study was supported by the China Science and Technology Innovation 2030- Major Project (No. 2022ZD0211701, 2021ZD0200700), the 10.13039/100014717National Natural Science Foundation of China (No. 82130042, 82471552), and Shenzhen Science and Technology Serial Funds (Guangdong, China) (No. GJHZ20210705141400002, KCXFZ20211020164543006, JCYJ20220818101615033, ZDSYS20220606100606014, and KQTD20221101093608028). The funding body had no role in the design of the study, data collection, analysis, interpretation, or manuscript writing.

## Conflict of interests

The authors declared no conflict of interests.
